# Paracrine Mechanisms of Mesenchymal Stromal Cells in Angiogenesis

**DOI:** 10.1155/2020/4356359

**Published:** 2020-03-09

**Authors:** Selma Maacha, Heba Sidahmed, Shana Jacob, Giusy Gentilcore, Rita Calzone, Jean-Charles Grivel, Chiara Cugno

**Affiliations:** ^1^Deep Phenotyping Core, Sidra Medicine, Doha 26999, Qatar; ^2^Advanced Cell Therapy Core, Sidra Medicine, Doha 26999, Qatar

## Abstract

The role of the mesenchymal stromal cell- (MSC-) derived secretome is becoming increasingly intriguing from a clinical perspective due to its ability to stimulate endogenous tissue repair processes as well as its effective regulation of the immune system, mimicking the therapeutic effects produced by the MSCs. The secretome is a composite product secreted by MSC *in vitro* (in conditioned medium) and *in vivo* (in the extracellular milieu), consisting of a protein soluble fraction (mostly growth factors and cytokines) and a vesicular component, extracellular vesicles (EVs), which transfer proteins, lipids, and genetic material. MSC-derived secretome differs based on the tissue from which the MSCs are isolated and under specific conditions (e.g., preconditioning or priming) suggesting that clinical applications should be tailored by choosing the tissue of origin and a priming regimen to specifically correct a given pathology. MSC-derived secretome mediates beneficial angiogenic effects in a variety of tissue injury-related diseases. This supports the current effort to develop cell-free therapeutic products that bring both clinical benefits (reduced immunogenicity, persistence *in vivo*, and no genotoxicity associated with long-term cell cultures) and manufacturing advantages (reduced costs, availability of large quantities of off-the-shelf products, and lower regulatory burden). In the present review, we aim to give a comprehensive picture of the numerous components of the secretome produced by MSCs derived from the most common tissue sources for clinical use (e.g., AT, BM, and CB). We focus on the factors involved in the complex regulation of angiogenic processes.

## 1. Introduction

Mesenchymal stromal cells (MSCs) were described for the first time in 1970 by Alexander Friedenstein as a “population of bone marrow stromal cells capable of mesodermal differentiation and trophic support of hematopoiesis” [1, 2]. Mesenchyme is derived from Greek, meaning “middle” (meso) “infusion,” and it refers to the ability of mesenchymatous cells to spread and migrate in early embryonic development between the ectodermal and endodermal layers [3]. MSCs are pluripotent, self-renewing, spindle-shaped cells found in several adult and perinatal tissues. To differentiate MSCs from other morphologically similar cells, the International Society for Cellular Therapy (ISCT) defined the minimal set of criteria by which MSCs are identified: adherence to plastic under normal cell culture conditions; differentiation capability into multiple cell lineages including, and not limited to, adipocytes, osteocytes, and chondrocytes; positive expression of CD105, CD73, and CD90 surface markers; and lack of expression of CD45, CD14, CD19, and CD34 and a minimal expression of HLA-DR [4]. MSCs can be derived from bone marrow (BM), adipose tissue (AT), and other adult tissues such as dental pulp and dermal tissues. MSCs can also be isolated from perinatal tissues such as cord blood (CB), placenta, and amniotic fluid and membrane (AM), as well as umbilical cord Wharton's jelly (WJ) [5–7].

MSCs possess therapeutic properties demonstrated both *in vitro* and *in vivo*, with evidence pointing to anti-inflammatory and immunomodulatory effects [8], as well as tissue regeneration, including healing of chronic wounds, regeneration of cartilage, angiogenesis, and vascularization following pathological conditions such as myocardial infarction, brain injury, and limb ischemia [9–14].

MSCs have generated considerable interest in the field of tissue regeneration and hold promising potential as a therapeutic approach due to their ability to enhance angiogenesis and accelerate tissue healing [15]. Indeed, angiogenesis is essential for tissue repair and an adequate vascular network is required to supply blood and growth factors to injured tissues. Although MSCs have been shown to play an important role in decreasing tissue damage and accelerating repair through the promotion of vascularization, the use of MSC-based therapies is restricted by their low level of persistence in targeted tissues and their limited capabilities of transdifferentiation *in vivo* [16–22]. While it was initially conceived that MSCs exerted their therapeutic effects by migrating to targeted sites of injury and actively contributed to tissue repair and regeneration, it is now increasingly acknowledged that MSCs do not typically engraft after transplantation, due to the phenomena of lung sequestration and systemic clearance [23, 24], and exhibit their therapeutic effect in a paracrine manner through the secretion of bioactive factors [13, 25, 26].

The paracrine effects of MSCs, firstly described by Gnecchi et al. [27], are due to numerous secreted elements collectively referred to as the secretome [28]. The secretome consists of all factors actively or passively released from cells; it contains soluble products composed of a proteic soluble fraction (mostly growth factors and cytokines) and a vesicular component, extracellular vesicles (EVs), which transfer proteins, lipids, and genetic material to recipient cells [29]. The MSC-derived secretome is very tissue- and/or individual cell-specific and is subject to fluctuations related to physiological states or pathological conditions. Moreover, the secretome is also affected by the preconditioning/priming of MSCs during cell culture prior to the collection of the conditioned media (CM) [10, 30, 31]. Thus, the appropriate therapeutic use of the MSC secretome as an active pharmaceutical ingredient as well as a drug delivery system [32] relies on the systematic quantitative and functional assessment of the MSC-secreted effectors from the perspective of specific clinical settings, e.g., macroareas such as angiogenesis, bone regeneration, and immune suppression.

**Figure 1:**
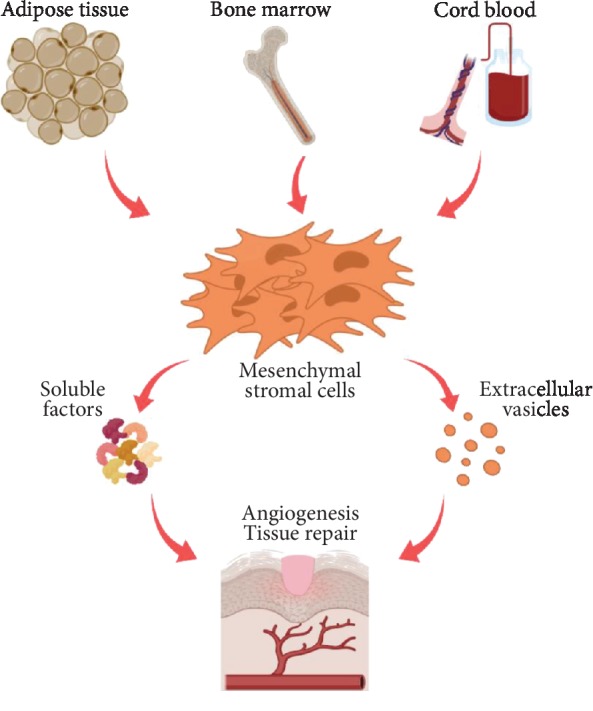
MSCs isolated and expanded from the most common sources (AT, BM, and CB) release their secretome *in vitro* and *in vivo* which acts upon mechanisms responsible for enhancing tissue repair and angiogenesis.

In the present review, the elements of the secretome of MSCs derived from the most common tissue sources for clinical use (e.g., AT, BM, and CB) will be explored in further detail addressing their roles in the angiogenic modulation (Figure 1), and data will be compared where available.

## 2. Role of Extracellular Vesicles

One paracrine mechanism of MSCs involves the secretion of EVs that have been shown to effectively mimic the therapeutic effects of MSCs, participating in tissue repair and regeneration in several preclinical models [33–35].

EVs are a heterogeneous population of cell-derived membrane vesicles that are secreted by almost all cell types including MSCs and serve as vehicles for bidirectional communication between cells [36]. Cells secrete a wide range of EVs that differ in size, origin, content, and function [37]. EVs include exosomes (also called small EVs), which are small membrane vesicles originating from the endocytic pathway, ranging from 30 to 150 nm in diameter and shed microvesicles (MVs, also called large EVs), which are large membrane vesicles of 150 to 1000 nm diameter budding off the plasma membrane [37]. The lipid bilayer of EVs encapsulates their bioactive contents (proteins, DNA, and RNA), protecting them from enzymatic degradation. Recently, it has become apparent that secreted EVs are proficient intercellular communication mediators through the transfer of their cargo to target cells and their ability to influence the behavior of recipient cells [36]. EVs can be purified from tissue culture supernatant as well as several biofluids (e.g., serum, plasma, saliva, ascites, cerebrospinal fluid, and urine). There is no general consensus as to the best purification method for EVs. The most common method for EV isolation is iodixanol density gradient ultracentrifugation, which separates vesicles according to their buoyant density by centrifugation. Size exclusion chromatography is also widely used for the isolation of EVs and separates vesicle particles based on their size. Immunoisolation could be a powerful method for the purification of EVs but requires the knowledge of established specific markers for EVs as well as tissue-specific discriminating markers. The International Society for Extracellular Vesicles (ISEV) has previously provided researchers with minimal experimental requirements for defining and assessing the quality/purity of isolated EVs in order to confidently report biological cargo or functions to EVs [38].

MSC-derived secretome performs its angiogenic modulation through a complex synergic activity between many bioactive molecules carried by EVs, such as microRNA (miRNA), transfer RNA (tRNA), long noncoding RNA (lncRNA), growth factors, proteins, and lipids [39].

### 2.1. EV-Associated Proteome

There is growing evidence that MSC-derived EVs play a significant role in the paracrine promotion of angiogenesis. Notably, MSCs may harbor a different angiogenic potential according to their tissue of origin. It has been reported that the AT-MSC-derived secretome displays a greater tubulogenic efficiency compared to BM-MSCs due to a higher expression of angiogenic factors such as insulin growth factor- (IGF-) 1, vascular endothelial growth factor- (VEGF-) D, and interleukin- (IL-) 8 [40]. Furthermore, comparative proteomic analysis of AT-MSC-derived EVs identified proteins that support a broad range of biological functions, including angiogenesis. The enriched angiogenic proteins identified in EVs comprise VEGF, von Willebrand factor (vWF), and transforming growth factor- (TGF-) *β*1 [41, 42]. Several functional studies investigating the paracrine actions of AT-MSCs have also involved EVs in modulating endothelial cell function and angiogenesis. For instance, it has been shown that AT-MSC-derived EVs induced *in vitro* vessel-like structure formation by human microvascular endothelial cells (HMEC) and that platelet-derived growth factor (PDGF) stimulated the secretion of EVs and further enhanced their angiogenic potential. Indeed, compared to unstimulated cells, PDGF-stimulated MSCs release EVs carrying angiogenic c-kit, stem cell factor (SCF), and matrix metalloproteinases (MMPs) [43]. Likewise, in a skin flap ischemia/reperfusion injury model, EVs were found to increase flap recovery and capillary density (increased tube formation) in a mechanism involving IL-6 [44]. Similarly, EVs, along with AT-MSCs, were also able to protect rat kidney from acute ischemia-reperfusion injury through the induction of angiogenesis, reflected by the increase of the angiogenic factors CD31, vWF, and angiopoietin [45].

In comparison to AT-MSCs, similar angiogenic effects were observed with other sources of MSCs such as BM or CB. For instance, Bian and colleagues have previously reported that upon hypoxia stimulation, BM-MSCs release EVs that could be quickly uptaken by HUVECs promoting their proliferation and migration along with tube formation *in vitro*. Similar observations were made *in vivo* using an acute myocardial infarction rat model, where intramyocardial injection of MSC-EVs was found to markedly enhance cardiac repair [46]. Likewise, EVs from BM-MSCs were shown to increase the proliferation and migration of fibroblasts derived from normal donors and diabetic chronic wound patients as well as tube formation by endothelial cells. Mechanistically, MSC-derived EVs were found to be enriched with transcriptionally active-signal transducer and activator of transcription 3 (STAT3), which was shown to control many aspects of angiogenesis by regulating VEGF expression at the transcriptional levels [47, 48]. Moreover, analysis of proangiogenic factors from BM-MSC-derived EVs capable of inducing endothelial cell migration through extracellular signal-regulated kinase (ERK)/Akt signaling revealed the presence of high levels of extracellular matrix metalloproteinase inducer (EMMPRIN) in these vesicles [49]. EVs derived from BM-MSC were also shown to carry VEGF and to activate VEGF receptors in endothelial cells leading to enhanced angiogenesis in ischemic limbs [50]. Furthermore, wingless-related integration site 3a (Wnt3a) was also described as a cargo of a subpopulation of EVs (CD63+) capable of inducing angiogenesis *in vitro* [51].

Several studies have reported that hypoxia potentiates the angiogenic potency of MSC-derived EVs, whether they are originating from AT, BM, or CB. Using a mouse model of subcutaneous fat grafting, hypoxic MSC-EVs from AT were capable of effectively promoting the survival of grafts and neovascularization (increased CD31-positive cells) [52]. Likewise, human umbilical vein endothelial cells (HUVECs) exposed to EVs from hypoxic AT-MSCs significantly upregulated angiogenesis-stimulating genes such as angiopoietin- (ANG-) 1 and VEGF receptor 2 [53]. Moreover, hypoxia not only induced the expression of VEGF in AT-MSCs and their EVs but also increased VEGF expression and protein kinase A (PKA) signaling pathway in HUVECs when exposed to hypoxia EVs [53].

Indeed, EVs derived from human CB-derived MSCs stimulated by hypoxia were also shown to promote angiogenesis both *in vitro* and *in vivo* [54–57]. Mechanistically, Wnt4 was identified in EVs derived from human CB-MSCs and was shown to induce *β*-catenin activation in endothelial cells leading to enhanced angiogenesis [58]. More recently, a proteomic analysis identified significant enrichment of hundreds of proteins in BM-MSC-derived EVs compared to parental BM-MSCs. Among these proteins, neuropilin 1 (NRP1) is interestingly known to regulate vasculogenesis, chemotaxis, migration, and invasion [59]. Such comprehensive proteomic techniques may be used to ultimately unravel the MSC-derived EV content and assess their angiogenic potential.

### 2.2. EV-Associated Nucleic Acids: miRNAs

About 98% of the entire genome is non-protein coding and was formerly termed as “junk DNA.” The noncoding DNA (ncDNA) is constituted of repetitive, transposable, interspersed elements, as well as noncoding RNA (ncRNA) genes. The ncRNAs encompass about 98% of the DNA transcript. Depending on their size, ncRNAs are arbitrarily distinguished into small (sncRNAs), if composed of less than 200 nucleotides (nt) (e.g., ribosomal RNA, transfer RNA, miRNA, small interfering RNA (siRNA), and piwi-associated RNA (piRNA)), and long (lncRNAs) if they have more than 200 nt [60]. The lncRNAs together with miRNAs, piRNAs, and endogenous siRNAs are involved in the epigenetic modification of DNA and in the regulation of transcriptional and posttranscriptional events.

In addition to growth factors and proteins, MSC-derived EVs were also reported to carry miRNAs. Since miRNAs are powerful regulators of gene expression, signaling through miRNA-EVs is a proficient paracrine mechanism used by MSCs to modulate angiogenesis. Regardless of the tissue of origin, enrichment of miRNAs in MSC-EVs has been shown to promote angiogenesis *in vitro* and *in vivo* (summarized in Table 1). miRNAs may act as suppressors of gene expression binding to the 3′-untranslated (3′-UTR) regions of specific mRNAs inhibiting their translation and/or promoting their degradation [61]. It has been reported that miRNAs target and modulate the expression of regulatory angiogenic genes encoding for cytokines, MMPs, VEGF, PDGF, fibroblast growth factor (FGF), and epidermal growth factor (EGF) [62]. For instance, miR-181b-5p was shown to modulate cerebral vascular remodeling after stroke. miR-181b-5p is carried in AT-MSC-derived EVs and enhances the mobility and angiogenesis of brain microvascular endothelial cells (BMECs) after oxygen-glucose deprivation by targeting transient receptor potential melastatin 7 (TRPM7) expression [63]. Several other miRNAs with angiogenic potential such as miRNA-494, miR-125a, or miR-210 were recently reported in MSC-derived EVs [64–66].

Moreover, MSC-EV delivery of miRNA-132, along with stromal cell-derived factor 1 (SDF-1), results in increased tube formation and enhanced angiogenic activity of endothelial cells in infarcted myocardial mouse models [67–69].

Furthermore, a recent comprehensive system study conducted by Ferguson and colleagues profiled the miRNAs contained in EVs isolated from human BM-derived MSCs and determined the dominant biological processes and pathways modulated by these miRNAs. Interestingly, around 23 miRNAs were identified as more abundant and were found to target genes related to cardiovascular and angiogenesis processes (more than 90 genes related to vasculature and tube development were targeted by miR-23a-3p, miR-424-5p, miR-144, and miR-130a-3p; and between 9 and 85 other cardiovascular development, angiogenesis, and tube formation genes were targeted by the remaining miRNAs). Functional testing of these EVs revealed that MSC-EVs were able to protect cardiomyocytes from apoptosis and increased angiogenesis in HUVECs [70].

### 2.3. Other EV-Associated Nucleic Acids

Unlike the parent cell, MSC-derived-EVs are highly enriched in the class of tRNAs (more than 50% of total small RNAs in AT-derived EVs and 23–35% in BM-derived EVs) [79]. tRNAs, along with piRNAs, contribute to maintaining stem cell potency [79], promoting cell survival and inhibiting cell differentiation of CB hematopoietic stem cells [80]. Their regulation of tissue healing and repair processes has been well-recognized [81, 82]. Stem cells can deliver ncRNAs to injured tissues through EVs, thus regulating specific programs of tissue regeneration [83]. As an example, MSCs can differentiate into endotheliocytes and myocytes in the mesoderm under conditional induction [84]. MSC-derived lncRNAs have been shown to support angiogenesis through the endothelial differentiation of MSCs: myocardial infarction-associated transcript (MIAT) targets miR200a and VEGF [85, 86]; maternally expressed gene 3 (MEG3) facilitates the ubiquitination and degradation of forkhead box protein M1 (FOXM1) reducing the angiogenic VEGF expression and promoting endothelial differentiation of MSCs [87].

### 2.4. Lipids

Lipids are also an integral part of EVs, and several studies have described EV lipid composition. EVs are rich in cholesterol, phospholipid (phosphatidylserine, phosphatidylcholine, phosphatidylethanolamine, and phosphatidylinositol), sphingomyelin, glycosphingolipids, diglyceride, polyglycerol, and ganglioside GM3 [88–90]. Specific lipids are enriched in EVs compared with their parent cells. Studies have shown a 2- to 3-fold enrichment from cells to MVs of cholesterol, glycosphingolipids, and phosphatidylserine [91–93] and a reduction of phosphatidylcholine [90].

As part of vesicle membranes, EV lipids provide the structural rigidity and stability required during the budding process as well as the ability to protect EV cargo, promoting autocrine or paracrine signaling [89, 94]. EV uptake can also be affected by lipid composition, with lipid rafts allowing the EVs to fuse into recipient cells [89]. As a result of EVs' high lipid content, they have an inherent capacity to pass through biological barriers and escape from phagocytosis by the reticuloendothelial system, while being biocompatible and immunologically inert [88, 95].

EVs released from different cell sources have distinct lipid content [92]. MSC-derived EVs are enriched in long lipid species and polyunsaturated acyl chains (more than 60 carbons and 10 double bonds) [92]; lysoderivatives of some phospholipids are enriched in large EVs and cardiolipin in small EVs. These characteristics are thought to allow for the curvature required and structural arrangement [92], although any biological functions are largely unknown. EVs are known to transport many bioactive lipids as well as lipid metabolism enzymes between cells. Possible bioactive lipids contained in MSC-derived EVs include leukotrienes, arachidonic acid, phosphatidic acid, prostaglandins, lysophosphatidylcholine, and docosahexaenoic acid [96]. In addition, the fate of EVs depends on the interaction of specific receptors with vesicular phosphatidylserine and lysophosphatidylcholine [97]. These interactions allow intercellular communications [98], chemoattraction [99], and apoptosis [100]. EVs derived from human BM-MSCs show an enrichment of sphingosine-1-phosphate (S1P), which is a signaling sphingolipid mediating cell proliferation, migration, and barrier function. When applied to chondrocytes, these EVs induce proliferation, matrix deposition, and cartilage defect repair. Blocking S1P reduced the therapeutic effect of MSC-derived EVs [91].

**Figure 2:**
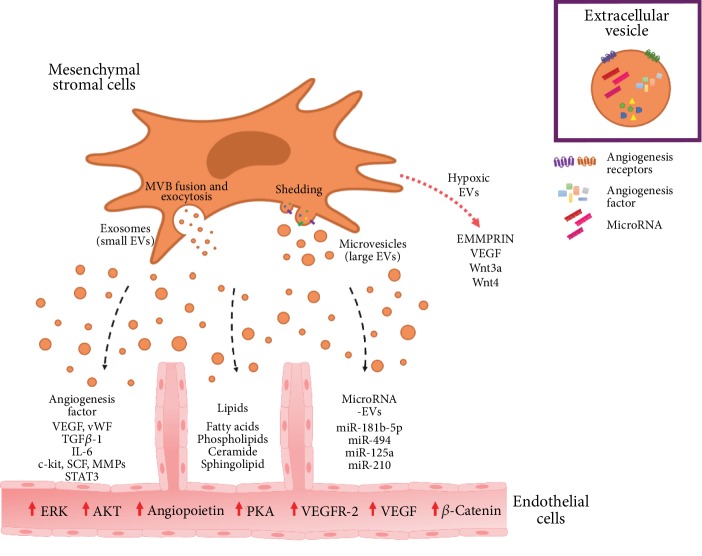
EV-mediated paracrine action of MSCs in angiogenesis. MSCs release EVs that are enriched in angiogenic factors such as cytokines, chemokines, and growth factors as well as miRNAs and lipids. Transferring of MSC-derived EV cargo to recipient endothelial cells triggers proangiogenic signaling important to tissue repair. In response to hypoxia, MSCs release EVs with an increased angiogenic potency capable of activating targeted signaling pathways to regulate the expression of angiogenic factors in endothelial cells.

The properties of MSC-derived secretome have also been studied after cell conditioning. Human BM-MSCs cultured in the presence of omega-6 and omega-9 fatty acids altered MSC expression and secretion of known mediators of angiogenesis, namely, the secretion of IL-6, VEGF, and nitric oxide. This indicates that fatty acids may affect MSC engraftment to injured tissue as well as MSC secretion of cytokines and growth factors that regulate local cellular responses to injury [101]. It has also been reported that MSCs lose their multipotency and undergo senescence during prolonged *in vitro* culture [102] and this can partly be attributed to a decrease in omega-6 fatty acids which in turn decreases membrane fluidity [103]. Supplementing lipids during the culture of human fetal membrane MSCs has been shown to increase cell proliferation rate, angiogenic differentiation, and immunomodulatory properties [104]. In addition, when these MSCs are exposed to EVs obtained from lipid-supplemented culture, cell migration rates improve as assessed by a wound-healing assay [105]. Depriving MSCs of serum and oxygen also alters its metabolic and lipidomic profile which in turn affects the composition of the released EVs. Under these conditions, higher ratios of lysophosphatidylcholine and phosphatidylethanolamine as well as ceramide are reported. Importantly, these lipids are associated with lipid rafts and involved in receptor-mediated intercellular signaling, respectively [106]. Taken together, these studies highlight the central role of the membrane system and bioactive lipids in cell physiology and angiogenesis. The effects exerted by MSC-derived EVs on endothelial cells are depicted in Figure 2.

## 3. Role of Soluble Proteins

MSCs release in the extracellular space a plethora of angiogenic factors including basic fibroblast growth factor (bFGF), VEGF, transforming growth factor-beta (TGF-*β*), PDGF, ANG-1, placental growth factor (PIGF), IL-6, hepatocyte growth factor (HGF), and monocyte chemoattractant protein 1 (MCP-1), which stimulate angiogenesis *in vitro* and *in vivo* [9, 107]. VEGF and TGF-*β*1 secreted in the CM promote angiogenesis and activate PI3K/Akt and MAPK pathways [9, 108]; HGF exhibits angiogenic properties on its own by inducing the expression of VEGF.

It has been reported throughout the literature that conditions such as exposure to tumor necrosis factor- (TNF-) *α*, interferon- (IFN-) *γ*, or hypoxia are able to modulate the composition of the MSC-derived secretome, a method generally referred to as priming. Hypoxia and serum deprivation priming are a driving factor towards the acquisition of a proangiogenic phenotype with MSCs derived from BM, AT, and placenta. They increase the production of VEGF, bFGF, HGF, IGF, and TGF-*β* [10, 30] and induce lipid and metabolic modifications within the cells and secreted EVs, as described above [106]. While the effects of culture conditions and priming on MSC-derived secretome are reported, the lack of standardization and regulation regarding culturing techniques prevents the definition of ideal culturing settings for producing the most effective secretome, from the source of MSCs used, the type of culture medium and complementary serums utilized, to the use of 3D cultures and scaffolding *vs.* more traditional 2D cultures.

The composition and concentration of angiogenic proteins vary between different MSC sources (WJ, AT, and BM), consequently affecting the functional responses. Recently, the angiogenic potential of WJ- and BM-derived MSCs has been described to be higher than AT-MSCs [108]. However, the paracrine activity of MSCs derived from neonatal tissues (CB and AM), when compared to adult tissues (AT), is not consistent across the different sources. An unbiased stable isotope labeling by amino acid- (SILAC-) based quantitative proteomic approach in cell culture coupled to LC-MS and validated by functional assays revealed that the secretome of MSCs isolated from fetal skin is superior to that of adult skin [109]. When considered across different sources, different patterns emerge: the secretion of VEGF and TGF-*β*1 is higher and comparable in AM-MSCs and AT-MSCs, while CB-MSCs show lower production, with an associated opposite trend for HGF (higher secretion in CB-MSCs *vs.* AM-MSCs) [110, 111]. On the other hand, Kim et al. showed increased secretion of VEGF-A, HGF, bFGF, and ANG-1 from amniotic-derived MSCs when compared to AT-MSCs [112].

Furthermore, macrophage colony-stimulating factor (M-CSF), interleukin-1 receptor antagonist (IL-1ra), and SDF-1a secretion were significantly higher in TNF-*α*- and IFN-*γ*-stimulated perinatal MSCs but lower than BM-MSCs while monocyte chemotactic protein-1 (MCP-1) was significantly higher in perinatal MSCs than BM-MSCs with no changes after stimulation [111].

The reports on which MSC source yields the most potent angiogenic and tubulogenic effects are sometimes conflicting. Hsiao et al. [40] reported that AT-MSCs are superior paracrine cells when compared to dermal tissue-derived and BM-MSCs due to heightened levels of VEGF, IGF-1, and IL-8, as well as MMP-3 and MMP-9 secretion. Du and colleagues paradoxically found that the CM of BM-MSCs and placental MSCs were more proangiogenic than those of AT-MSCs and umbilical cord-derived MSCs [113].

bFGF is not consistently found in the secretome regardless of the MSC source, even after stimulation with TNF-*α* and IFN-*γ* [111]. Moreover, the chemotactic factor growth-related oncogene (GRO) has been detected at low levels in BM-MSCs but not in AT-MSCs or dermal-derived-MSCs [40]. Likewise, MCP, RANTES, SCF, and SDF-1 have shown variations in the CM of MSCs of different origins, while high levels of IL-6 are usually constantly retrieved [40].

Among all VEGF family components, VEGF-C and VEGF-D represent the major factors for angiogenesis and lymphangiogenic processes [114–116]. VEGF-D is expressed at higher levels in AT-MSCs compared to other MSCs [40] and is upregulated by hypoxia in BM-MSCs [114]. The Akt/Nrf2 pathway plays a crucial role in promoting the angiogenic-related functions of BM-MSCs [117], as demonstrated during infarction, where MSCs overexpressing AKT1 are able to preserve normal pH levels in the surviving myocardium [118]. FGF-2 modulates angiogenesis with autocrine mechanism [119], together with VEGF, has a potent synergistic effect on the induction of angiogenesis *in vivo* [120], and supports long-term angiogenic efficacy of AT-MSCs in ischemic mouse tissues [121]. A comparative MSC-derived secretome profile relevant to angiogenesis is provided in Table 2. Finally, the angiogenic activity mediated by the secretome can be significantly inhibited by neutralizing antibodies against specific cytokines, such as VEGF, MCP-1, and IL-6 [9]. The MSC-derived secretome therapeutic potential to accelerate angiogenesis is extensively reviewed by Bronckaers et al. [122].

In addition to antibody-based techniques assessing known angiogenic factors present in the secretome of MSC, the field is in need of more exhaustive proteomic techniques. Indeed, a comprehensive analysis of the MSC-derived secretome is an essential step to a better understanding of the therapeutic components of MSCs. To date, mass spectrometry represents a powerful high-throughput technique enabling the identification and quantification of thousands of proteins present in the MSC secretome [108, 123, 124]. Taking advantage of such high-throughput proteomic techniques will certainly improve the characterization of MSC secretome and help to judiciously implement MSC-based therapeutic products in the clinic.

## 4. Limited Clinical Studies

**Figure 3:**
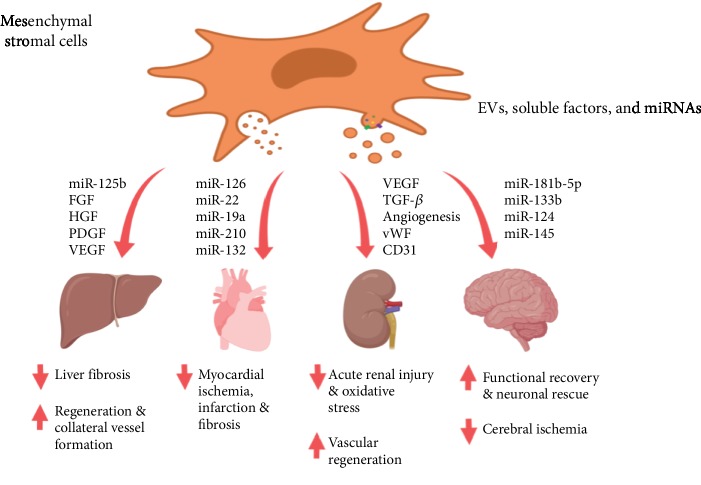
Schematic representation of the secretome effects on major organs *in vivo*. EVs, soluble factors, proteins, and miRNAs present in the MSC secretome are in favor of regeneration, reperfusion, and recovery of major organs and tissues following trauma and ischemic injury.

The MSC secretome represents a promising angiogenic therapy option for treating ischemic diseases (e.g., coronary vascular disease, cerebral infarction, and limb ischemia), neurodegeneration, wound healing, tissue/organ fibrosis, etc. [125] (Figure 3 provides schematic representation). However, until now, it has only been studied and applied in experimental models, though numerous.

The sole clinical trial currently registered on http://clinicaltrial.gov/ is the NCT03384433 for the use of allogenic MSC-small EVs in patients with acute ischemic stroke, which however did not recruit patients so far. Nonetheless, one limited clinical report has been published in a different clinical setting (refractory graft versus host disease) [126].

Challenges and constraints in clinical-grade production of MSC secretome, choice of MSC source (likely to be specifically addressed per each disease setting), and preconditioning have been recently reviewed by Teixeira and Salgado [127].

## 5. Conclusions

Therapeutic angiogenesis depends on the efficient delivery of exogenous angiogenic factors to stimulate neovasculature formation. MSC-derived secretome seems to mediate beneficial angiogenic effects in a variety of tissue injury-related diseases, therefore supporting the development of cell-free therapeutic products that exert similar desirable effects to systemic MSC infusion therapy. Interestingly, MSC-derived secretome as a cell-free therapy bypasses the risks associated with stem cell-based therapies, namely, immune-mediated rejection, accumulation of genomic alterations, senescence-induced genetic instability, and complicated safety regulatory setting [128–131].

The expected successful clinical application of MSC-derived secretome will require a comprehensive understanding of its different components, the *in vivo* functionality of the proteins secreted, and their specificity to a given pathological setting. Definitely, a major cause of failure of MSC-based therapeutic products is the incomplete characterization of their features and activities. The successful clinical application of MSC-secreted factors will require many more comprehensive studies using an “omics” approach, including quantitative proteomic assessment of different MSC populations of clinical relevance. Although comprehensive and quantitative information on proteins through mass spectrometry can appear more challenging (compared to gene expression or antibody-based assays), high-throughput proteomic techniques are greatly needed for deciphering the protein make-up of MSCs as well as quantifying the changes in physiological or pathological conditions. New technological and bioinformatics advances in mass spectrometry bring this closer to reality [132]. Moreover, the development of standardized procedures for large-scale clinical-grade secretome-based products and their manufacturing, which is highly needed for future clinical trials, remains insufficient. Therefore, we believe that the current resources dedicated to MSC-related regenerative therapies should be better exploited to unveil the features and functionality of the MSC secretome and to enable a GMP-standardized production of ready-off-the-shelf, well-characterized acellular products. This investment is indeed more likely to pay back by providing a stable therapeutic strategy which is well characterized than using MSCs as cell products.

## Figures and Tables

**Table 1:** Extracellular vesicle miRNA cargo and MSC-mediated angiogenesis.

MicroRNA	MSC (tissue of origin)	Function	Reference
miR148a, miR532-5p, miR378, let-7f	Porcine adipose tissue	Putative tissue regeneration by inducing several cellular pathways including angiogenesis	[71]
miR494	Human bone marrow	Muscle regeneration by enhancing myogenesis and angiogenesis	[64]
miR-19a	Rat bone marrow (GATA-4-overexpressing MSC)	Cardioprotection by increasing survival and angiogenesis	[72, 73]
miR-125a	Human adipose tissue	Induction of angiogenesis through the repression of DLL4 expression	[65]
miR-210	Mouse bone marrow	Promotion of angiogenesis in a mouse myocardial infarction model through the repression of Efna3 expression in endothelial cells	[66, 74]
miR-30b	Not available	Promotion of angiogenesis *in vitro* and *in vivo*	[75]
miR-21a-5p	Mouse bone marrow	Cardioprotection through inhibition of proapoptotic genes; *in silico* data support a role in angiogenesis	[76]
miR-210-3p	Mouse bone marrow	Acceleration of recovery of hindlimb ischemia through the promotion of angiogenesis	[50]
miR-31	Human adipose tissue	Promotion of angiogenesis in HUVECs by targeting the antiangiogenic HIF-1 gene	[77]
miR-181b	Rat adipose tissue	Promotion of the mobility and angiogenesis of brain microvascular endothelial cells (BMECs) after oxygen-glucose deprivation (OGD) through the repression of TRPM7 expression	[63]
miR-21-5p	Human endometrium	Cardioprotection and enhancing microvessel density in a rat model of myocardial infarction	[78]

**Table 2:** Angiogenic secretome of MSCs from different sources.

Angiogenic factors	High	Low	None	Ref
Angiogenic potential	WJ-MSC, BM-MSC, placenta-MSC	AT-MSC, umbilical cord		[108] [114]
VEGF secretion	AM-MSC, AT-MSC	CB-MSC, BM-MSC		[112] [40]
TGF-*β*1 secretion	AM-MSC	AT-MSC		[112]
VEGF-A, HGF, bFGF, ANG-1	AM-MSC	AT-MSC		[113]
M-CSF, IL-1ra, SDF-1*α*	Primed perinatal MSCs	BM-MSCs		[112]
MCP-1	Perinatal MSCs	BM-MSCs		[112]
IGF-1, IL-8, MMP-3, MMP-9	AT-MSC	BM-MSC, D-MSC		[40]
GRO		BM-MSCs	AT-MSCs, D-MSCs	[40]
IL-6	MSCs			[9] [108]
VEGF-D	AT-MSC, BM-MSCs			[40, 113]
FGF-2	AT-MSC			[120]
